# Nitrites and nitrates dietary exposure from natural sources and additives and type-2 diabetes risk

**DOI:** 10.1093/eurpub/ckac129.490

**Published:** 2022-10-25

**Authors:** B Srour, E Chazelas, C Debras, N Druesne-Pecollo, C Agaesse, F Szabo de Edelenyi, L Sellem, E Kesse-Guyot, M Deschasaux-Tanguy, M Touvier

**Affiliations:** EREN, Inserm, Inrae, Cnam, USPN, INSERM, Bobigny, France

## Abstract

Nitrates and nitrites occur naturally in water and soil and are commonly ingested from drinking water and dietary sources. They are also used as food additives. The epidemiological evidence linking exposure to nitrites/nitrates with type-2 diabetes (T2D) risk is scarce. We aimed to study these associations in a large population based prospective cohort study. Overall, 104,168 adults from the French NutriNet-Santé cohort study (median follow-up time 6.7 years) were included. Associations between intakes of nitrites and nitrates (evaluated using repeated 24h dietary records, linked to a comprehensive food composition database and accounting for details of commercial names/brands of industrial products) and risk of T2D were assessed using cause-specific multivariable Cox proportional hazard models adjusted for known risk factors (sociodemographic, anthropometric, lifestyle, medical history, and nutritional factors). During follow-up, 969 incident T2D cases were ascertained. Total nitrites and nitrites from natural sources were both positively associated with higher T2D risk (HRtertile 3 vs.1=1.29 (95% CI 1.06-1.56), Ptrend=0.004, and 1.27 (95% CI 1.05-1.54), Ptrend=0.01, respectively). Participants with higher exposure to nitrites from food additives (i.e. above the sex-specific median), and specifically those having higher exposure to sodium nitrite (e250) had a higher T2D risk compared with those who were not exposed to food additive nitrites (HRtertile 3 vs.1=1.58 (95% CI 1.28-1.94), Ptrend<0.001, and 1.59 (95% CI 1.30-1.96), Ptrend<0.001), respectively). There was no evidence for an association between nitrates of any source and T2D risk (all Ptrend>0.4). In this large prospective cohort, a higher dietary exposure to nitrites (from both natural sources and food additives) was associated with higher T2D risk. These results provide additional evidence in the context of current discussions about updating regulations on the use of nitrites as food additives.

**Key messages:**

• A high exposure to dietary nitrites (from both natural and food additive sources) is associated with an increased risk of type-2 diabetes.

• These findings support further regulations concerning the use of nitrites as food additives in processed meats.

